# Interferon-γ release assay as a sensitive diagnostic tool of latent tuberculosis infection in patients with HIV: a cross-sectional study

**DOI:** 10.1186/s12879-018-3508-8

**Published:** 2018-11-19

**Authors:** Giselle Burlamaqui Klautau, Nadijane Valéria Ferreira da Mota, Mauro José Costa Salles, Marcelo Nascimento Burattini, Denise Silva Rodrigues

**Affiliations:** 1 Division of Infectious Diseases, Department of Internal Medicine Santa Casa de São Paulo School of Medical Sciences, Hospital da Irmandade da Santa Casa de Misericórdia de São Paulo, Rua Dr Cesáreo Mota Jr 112,, São Paulo, SP CEP: 01303-060 Brazil; 2 Clemente Ferreira Institute, Rua da Consolação 717, São Paulo, SP CEP: 01221-020 Brazil; 3 Emílio Ribas Institute of Infectious Diseases, Av Dr Arnaldo 165, São Paulo, SP CEP: 01246-900 Brazil; 4 Federal University of São Paulo (UNIFESP), Rua Sena Madureira 1500, São Paulo, CEP: 04021-001 Brazil

**Keywords:** HIV, Latent tuberculosis infection, Tuberculin skin testing, Interferon-γ release assay, AIDS

## Abstract

**Background:**

In developing countries, tuberculosis (TB) is a major public health problem and the leading cause of death among patients with HIV (Human Immunodeficiency Virus). Until 2001, the tuberculin skin test (TST) was the only available tool for the diagnosis of latent tuberculosis infection (LTBI), but false-negative TST results are frequently reported. Recently, the interferon-γ (IFN-γ) release assay (IGRA) has gained ground because it can detect the IFN-γ secreted by circulating lymphocytes T cells when stimulated by specific TB antigens. However, the role of IGRA in the diagnosis of LTBI in HIV-infected patients has not been well established.

**Methods:**

This cross-sectional study compared the accuracy of TST (performed by the Mantoux method) and IGRA (QuantiFERON-TB Gold In-Tube, Cellestis, Carnegie, Australia) on the diagnosis of LTBI among patients with HIV. LTBI is defined by LTBI risk and at least one positive test (TST or IGRA), without clinical evidence of active TB. We also assessed the accuracy of TST and IGRA among HIV patients with high and low risk for LTBI.

**Results:**

Among 90 HIV patients, 80 met the study criteria for LTBI, fifty-nine (73.7%) patients were TST positive, 21 (26.2%) were negative, whereas 75 patients (93.7%) were IGRA positive, and five (6.2%) were negative. TST showed poor agreement with the diagnosis of LTBI (Kappa: 0.384), while IGRA demonstrated good agreement (Kappa: 0.769). Among 69 patients with high risk and 21 with low risk for LTBI, TST was positive in 48 (69.5%) and 11 (52.4%), while IGRA was positive in 68 (98.5%) and 7 (33.3%) patients, respectively. There were no association between TST and the level of risk (*P* = 0,191). Conversely, we observed a strong association between the IGRA and risk for LTBI (*p* < 0.001).

**Conclusions:**

Compared to TST, IGRA positivity is consistent with the risk of TB infection and seems to be a better diagnostic tool for LTBI in HIV-infected patients.

**Electronic supplementary material:**

The online version of this article (10.1186/s12879-018-3508-8) contains supplementary material, which is available to authorized users.

## Background

One-quarter of the world population is estimated to be infected with *Mycobacterium tuberculosis*, while 10.0 million cases of TB occurred worldwide in 2017 [1–3]. The best estimate is that there were 1.3 million TB deaths in 2017, with an additional 300.000 deaths resulting from TB disease among the population living with HIV [3]. TB is a major public health problem in Brazil and the leading cause of death in patients with AIDS and one of the most common opportunistic infections [4, 5]. Moreover, recently published data collected in the city of São Paulo found that among 78.6% of patients diagnosed with tuberculosis who underwent HIV testing, 9.9% were coinfected [6].

A significant number of active TB cases arise in people with LTBI within a period of 2–5 years following primary infection [7]. Between 5 and 15% of individuals with LTBI progress to active TB, and the risk of active TB increases with poor immunity, reaching 30% among individuals infected with HIV [3, 8, 9].

The available tests for the diagnosis of latent TB infection (LTBI) have limitations that pose great challenges for both the diagnosis and the treatment of these patients [10, 11]. Indeed, the diagnosis of LTBI is a worldwide problem due to the lack of a gold standard. In countries with a high incidence of tuberculosis, including Brazil, the diagnosis of LTBI has been based upon a positive TST test in individuals in whom TB disease has been ruled out [4, 5]. The TST is an easy and low-cost test to apply, although, its specificity may be affected by previous BCG (Bacillus Calmette-Guérin) vaccination or even by infection with nontuberculous mycobacteria [12]. In addition, false-negative TST results can be observed in anergic or immunodeficient patients, including individuals with AIDS [13].

Over the last years, IGRA has gained ground as an alternative to the TST because it can detect the IFN-γ secreted by circulating lymphocytes T cells when stimulated by specific TB antigens. It can be measured in the blood and used as a tool for the diagnosis of LTBI [14–17]. Studies comparing the use of the IGRA and TST for the diagnosis of LTBI have shown that the IGRA has higher specificity, especially among populations submitted to BCG vaccination [18]. Nevertheless, the TST and IGRA both have suboptimal sensitivity, and conflicting results are common. It has been described that TST have particularly low sensitivity when testing immunocompromised individuals for LTBI, and thus might not be an adequate diagnostic tool applied for patients with HIV [16]. Conversely, the IGRA seems to be highly specific for the detection of LTBI, especially in BCG-vaccinated individuals and may help to identify LTBI in HIV-infected immunosuppressed patients [17].

The detection of LTBI and the treatment of patients at increased risk for developing active TB are the only effective strategies to prevent the development of tuberculosis [3, 5, 8]. Since validated guidelines for the proper diagnosis of LTBI in HIV patients are lacking, we hypothesized that applying IGRA would improve the diagnosis of LTBI compared to that with conventional TST test in adults infected with HIV/AIDS. Furthermore, we assessed the accuracy of TST and IGRA in HIV-infected patients presenting higher and lower risk for LTBI.

## Methods

This work was a cross-sectional study of 90 HIV-infected individuals older than 18 years, at risk for LTBI and recruited between March 2012 and April 2013, at the Santa Casa de São Paulo School of Medical Sciences, Clemente Ferreira Institute, and Emílio Ribas Institute of Infectious Diseases in São Paulo, Brazil. Patients diagnosed with mycobacteria other than TB, as well as patients with other known causes of immunosuppression, including type 1 diabetes, cancer, and/or the use of immunosuppressive medications, were excluded from the study. This study was approved by the ethics committees of all three institutions involved with this study. Only those individuals who agreed to participate and who signed the informed consent form were included in the study.

### Definition and risk of LTBI

Latent tuberculosis infection (LTBI) is characterized by the presence of immune response to previously acquired *Mycobacterium tuberculosis* infection without clinical evidence of active tuberculosis [19]. LTBI was defined as those with a definite risk of LTBI (high or low) with at least one positive test (TST or IGRA), in which tuberculosis disease was absent (clinical manifestations of TB and/or radiological signs suggesting TB), and with smear-negative for tuberculosis in at least two sputum samples. The LTBI risk definition was established according to previous studies [4, 20–22].

High risk patients for LTBI was defined based upon extensive period of household contact with a smear-positive pulmonary tuberculosis person, which may have occurred during nocturnal as well as extensive diurnal periods. In addition, TB index-case patients should not have started TB treatment and presented signs and symptoms of active disease, including cough longer than 3 weeks plus at least one of the following: (a) losing 10% of body weight, (b) fever (> 38 °C), and (c) night sweats. Individuals with low risk were those with outside household contact history not sharing the domicile, but instead they share the same physical space at work, during educational or social activities with the index case. In addition, index case should not have started TB therapy and presented with only one of the following signs or symptoms of the index case: cough (> 3 weeks), fever (> 38 °C), sweating or weight loss (> 10% of body weight). The principal distinction between high and low risk patients for LTBI was presence of household contact and cough in the high-risk group.

The main diagnosis criteria for active TB in the index case was microbiology; presence of sputum with positive direct bacilloscopy (by Ziehl-Neelsen staining), culture in Lowenstein-Jensen medium or an anatomopathological examination showing caseating granulomas and acid-fast bacilli in tissue specimens. The lung tissue fragments used in the anatomopathological study were obtained by means of transbronchial biopsy. Negative microbiological results on smears or lung tissue cultures were excluded despite the presence of cavities on chest x-ray. Individuals who were double-negative for TST and IGRA were considered LTBI-free.

### Tuberculin skin test (TST) and interferon-γ release Assy (IGRA) with QuantiFERON®-TB gold in-tube (QFT-GIT)

#### Tuberculin skin test (TST)

TST was performed using the Mantoux method [23], with intradermal administration of 0.1 ml purified protein derivative (0.1 ml tuberculin PPD RT23 2 TU, SSI, Copenhagen, DK) on the middle third of the anterior face of the left forearm. The reading occurred 48–96 h after the application, using the palpation method of the maximum transverse diameter of the induration and using a ruler in millimeters for measurement according to the National Health Foundation recommendations. For all individuals who participated in the study, it was considered a reactive when the size of the induration was ≥5 mm.

#### Interferon-γ release assay (IGRA) with QuantiFERON®-TB gold in-tube (QFT-GIT)

The IGRA was performed according to the manufacturer’s recommendations (QFT-GIT manufacturer: Cellestis, Carnegie, Australia). Blood samples for IGRA testing were collected and the blood samples were transported to the Instituto Clemente Ferreira (ICF, São Paulo, Brazil). An aliquot of 3.0 ml of blood was withdrawn by trained technicians. Three tubes were marked with the same identification number of the patient’s questionnaire: negative control bottle (gray cap), vial coated with tuberculosis-specific antigens (red cap) (ESAT-6, CFP-10, TB7.7 [p4]) and positive control vial coated with phytohemagglutinin as mitogen (purple cap). After collection and homogenization, tubes containing blood were incubated at 37 °C for 16–24 h and kept upright during this period. After 16 to 24 h the tubes were centrifuged and then the plasma was separated and frozen at − 70 °C. When the number of patients required to perform an ELISA plate (29 patients) or two plaques (58 patients) was reached, the ELISA assay was performed. Each patient had their plasma sample evaluated for the production of interferon gamma by lymphocytes after stimulation by specific antigens of the *M. tuberculosis*, inert antigens (negative control) and mitogens (positive control). After obtaining the ELISA crude values, these were analyzed from a specific software for QuantiFERON®-TB Gold In Tube and the results further calculated. The program evaluates the quality of the analyzes, generates a standard curve and provides a result for each individual. The final test result may be positive or negative. Those with antigen TB values ​​minus Nil (TB Ag-Nil) greater than or equal to 0.35 IU/ml are considered positive. In the study, the “undetermined” result of the IGRA was considered as “positive”.

Treatment for LTBI was recommended to individuals who were positive for either TST or IGRA and for whom TB disease was ruled out, following the standards of the Brazilian Ministry of Health [4, 5].

### CD4+ and CD8+ cell counts

For this study, we considered T lymphocyte subpopulations CD4 + and CD8 + counts, obtained by flow cytometry up to three months before the data collection [24].

### Statistical analysis

To investigate whether an association exists between the TST and the IGRA, we used the McNemar test. If agreement between variables was found, then we used the Kappa value to determine the level of agreement. To evaluate the values of CD4 + T cells according to the result of the TST and IGRA, we used the Mann-Whitney test. To examine the association between the test results and the risk of LTBI, we used Fisher’s exact test, the McNemar test (if there was agreement), and the Kappa value, if needed, to determine the degree of agreement [25]. Receiver operating characteristic (ROC) curve was used to verify the possibility of identifying cut-off values of CD4+ T and CD8+ T cells, to optimize the sensitivity and specificity of the test results in accordance with the risk of LTBI. It was also plotted to compare the diagnostic accuracy of tests. We defined the following agreement levels: no correlation between the variables when the Kappa value was less than 0.20; poor or fair agreement for values from 0.21 to 0.40; moderate agreement for values from 0.41 to 0.60; good agreement for values from 0.61 to 0.80; and strong agreement for values from 0.81 to 1. A significance level of 5% (0.05) was adopted throughout the study. We used the statistical software Predictive Analytics Software 17.0.2 (PASW Statistics 17.0.2).

## Results

Initially, 106 patients were considered eligible for the study. Of these, 16 were excluded, three for not agreeing to sign the consent form, five for not returning for TST reading and eight for lack of recent CD4 + T lymphocyte count and HIV viral load (last three months before the data collection). Therefore, 90 HIV-infected patients at risk of latent tuberculosis infection were included in the analysis, of which 56.7% were male with a mean age of 39.2 (± 10.9) years. Most of them (93.3%) were under antiretroviral therapy and 95.5% had been previously vaccinated for tuberculosis with BCG vaccine. Those classified at high and low risk for TB infection were 69 (76.6%) and 21 (23.3%) patients, respectively. Other clinical characteristics of the study population are shown on Table 1. The median values of CD4 + and CD8 + T cells, and HIV viral load are described in Table 2. Briefly, CD4 + T cells median value was of 557.5 (± 283.89) cell /mm^3^, and mean HIV viral load values was 514.5 (± 1814.02) copies/ml.

**Table 1:** Demographic information of the studied population of 90 HIV-infected patients at risk of LTBI

	Frequency (*n*)	Percentage(%)
Sex		
Female	39	43.3
Male	51	56.7
BCG vaccination		
No	04	4.4
Yes	86	95.5
BCG scar		
No	04	4.4
Yes	86	95.5
Comorbidities		
No	36	30
Yes	54	60
ART		
No	6	6.6
Yes	84	93.3
Risk of infection for TB		
High	69	76.6
Low	21	23.3

*HIV* Human immunodeficiency vírus, *LTBI* Latent tuberculosis infection, *BCG* Bacillus Calmette-Guérin, *ART* antiretroviral therapy, *TB* Tuberculosis

**Table 2:** Median values of CD4+ and CD8+ T lymphocyte counts, CD4/CD8 values, HIV viral load, and log^10^ of HIV viral load in 90 patients with HIV at risk of LTBI

Variables	Median	SD	Minimun	Maximun
CD4+	557.5	283.8	114.0	1259.00
CD8+	835.4	331.7	225.0	2112.0
CD4+/CD8+	0.71	0.43	0.16	3.53
HIV (VL)	514.5	1.814	50.0	10,023.0
HIV log^10^(VL)	1.89	0,55	1.70	4.00

*CD4+* Cluster of differentiation – 4, *CD8+* Cluster of differentiation – 8, *HIV* Human Immunodeficiency virus, *VL* viral load, *SD* Standard Deviation

### TST and IGRA results

Among 90 HIV patients, 10 patients were TST and IGRA tests negative and therefore did not meet the study criteria for LTBI. However, we could not tell whether these individuals were truly negative for LTBI or whether they were false-negative individuals. Among them, average age was 41 years, the average time of active TB exposure to infected individuals was 1.7 months, and the average CD4+ T cell count ​​was 415 cells/mm^3^. All patients were receiving ART and had undetectable viral load, hence were most likely not to be infected with TB.

Table 3 summarizes the frequencies of TST and IGRA results in 90 HIV-infected patients included in the study. Briefly, among 80 patients who met the study criteria for LTBI, 59 (73.7%) were TST positive, while 21 (26.2%) were negative. In contrast, 75 (93.7%) patients with HIV and with LTBI were IGRA positive, and only five patients (6.2%) were negative. When assessing the level of agreement between TST and IGRA with the diagnosis of LTBI, TST showed poor agreement (Kappa: 0.384), while the IGRA showed good agreement (Kappa: 0.769).

**Table 3:** Description of frequencies related to the TST and IGRA result, according to diagnosis for LTBI in 90 HIV-infected studied

Test type	Results	Diagnosis of LTBI (*n*)		Total
		No	Yes	
TST	Negative	10	21	31
	Positive	0	59	59
	Total	10	80	90
IGRA	Negative	10	5	15
	Positive	0	75	75
	Total	10	80	90

*TST* Tuberculin Skin Test, *IGRA* Interferon-γ release assay, *LTBI* Latent tuberculosis infection, TST and LTBI (Kappa: 0.384), IGRA and LTBI (Kappa: 0.769)

Interestingly, only one patient presented indeterminate IGRA result, but with TST positive. This patient had a normal CD4 + T lymphocyte count (938 cels/mm^3^).

**Fig. 1:**
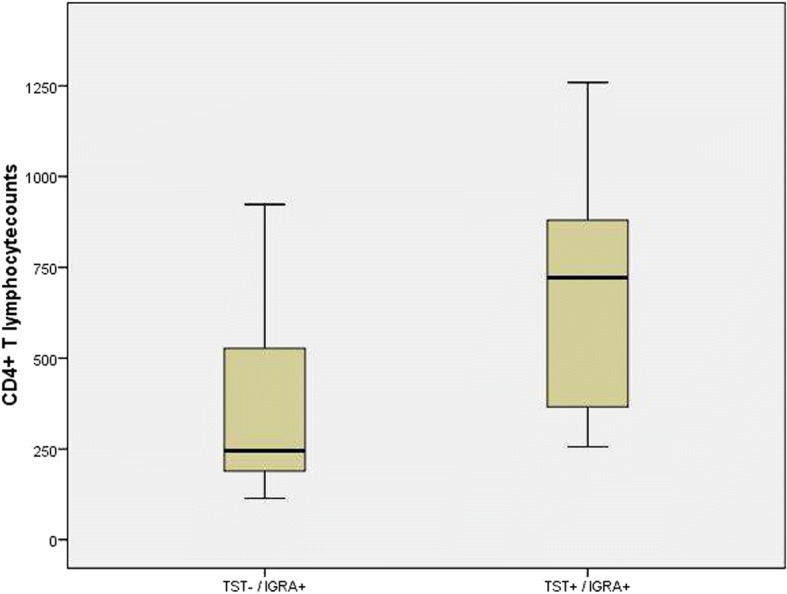
CD4+ T lymphocyte counts and TST outcome in 90 HIV-infected patients with positive IGRA results and at risk of LTBI. CD4+: Cluster of differentiation – 4; HIV: Human Immunodeficiency virus; TST: Tuberculin Skin Test; IGRA: Interferon-γ release assay; LTBI: Latent tuberculosis infection

Regarding discordant results between tests, 21 patients presented negative TST and positive IGRA. These patients had an average CD4 cell count of 245 cells/mm^3^, while patients with both TST and IGRA positivity (*n* = 54) had an average CD4 cell count of 721.5 cells/mm^3^. Indeed, the average CD4 cell counts in the group presenting TST negative and IGRA positive were significantly lower than in the double-positive (TST and IGRA) group (*p* < 0.001) (Fig. 1).

Among 69 (76.7%) patients presenting high risk for TB infection, TST was positive in 48 (69.5%), negative in 21 (30.5%), respectively. No significant association was found between the TST and the level of risk (*p* = 0.191). On the other hand, IGRA was positive to all but one patient (98.5%). We observed a strong association between the IGRA and risk for LTBI (*p* < 0.001). Low risk patients were 21 (23.3%), of which TST was positive in 11 (52.4%) and negative in 10 (47.6%), respectively. Conversely, IGRA was positive in seven (33.3%) and negative in 14 (66.7%) patients. Hence, TST results were not correlated with risk for TB infection, whereas a good correlation was found between IGRA and risk (Table 4). Using a threshold value of 0.51UI/mL for the IGRA yielded a ROC curve with 98.6% of sensitivity and 71.4% of specificity (see Additional file 1). We observed that only six patients at low risk of LTBI were positive for both TST and IGRA, while among patients at high risk, 48 out of 69 patients (69.6%) had double-positive results (TST and IGRA). The sensitivity (Se), specificity (S), positive predictive value (PPV), and negative predictive value (NPV) were estimated for both the TST and the IGRA, considering the risk as the main criterion for a diagnosis of LTBI (Table 5).

**Table 4:** Description of frequencies related to the TST and IGRA result, according to the risk for LTBI in 90 HIV-infected patients studied

Test type	Results	Risk		Total
		High	Low	
TST	Positive	48	11	59
	Negative	21	10	31
	Total	69	21	90
IGRA	Positive	68	7	75
	Negative	1	14	15
	Total	69	21	90

*TST* tuberculin skin test, *IGRA* Interferon-γ release assay, *LTBI* Latent tuberculosis infection; TST and risk (Fisher, *p* = 0.191). TST and risk (Kappa: 0.147, 90% CI: 0.000–0.315); IGRA and risk (Fisher, *p* < 0.001); IGRA and risk (Kappa: 0.724, 90% CI: 0.555–0.894)

**Table 5:** Comparison between TST and IGRA in 90 patients infected with HIV at risk of LTBI

Test	Measure	Estimate	CI 95%	
TST	Se	69.57	57.31	80.08
	S	47.62	25.71	70.22
	PPV	81.36	69.09	90.31
	NPV	32.26	16.68	51.37
IGRA	Se	98.55	92.19	99.96
	Es	66.67	43.03	85.41
	PPV	90.67	81.71	96.16
	NPV	93.33	68.05	99.83

*HIV* Human Immunodeficiency virus, *TST* Tuberculin Skin Test, *IGRA* Interferon-γ release assay, *CI* confidence intervals, *Se* sensitivity, *S* specificity NPV, negative predictive value, *PPV* positive predictive value

Discordant results between TST and IGRA performed in 90 HIV-positive patients under the risk for LTBI are described in Table 6. Briefly, 21 patients presented negative TST and positive IGRA.

**Table 6:** Description of the frequencies related to the association of the TT and IGRA results in 90 HIV-infected patients at risk of LTBI

	TST		
	Negative	Positive	Total
IGRA			
Negative	10	5	15
Positive	21	54	75
Total	31	59	90

*TST* Tuberculin Skin Test, *IGRA* Interferon-γ release assay, *LTBI* Latent Tuberculosis Infection, TST and IGRA (McNemar, *p* = 0,003). TST and IGRA (Kappa: 0,271, 90% IC: 0,116-0,426)

## Discussion

In the present study, we compared the TST with QFT-GIT (IGRA) to establish which test would be most accurate for the diagnosis of LTBI in HIV-infected adults. We acknowledge that most of HIV patients analyzed were under antiretroviral therapy (ART) and consequently had adequate immunological and virologic control of the disease showing high levels of CD4+ lymphocytes (> 500 cells/mm^3^). This situation constitutes the ideal timing for the diagnosis and treatment of LTBI [26].

Nonetheless, most of the studied patients were at high risk of LTBI. Categorizing patients with HIV into high or low risk may be justified by the poor performance of the TST for the diagnosis of LTBI, although it remains an important diagnostic tool in countries with a high prevalence of TB [7]. The lack of a gold standard suitable for the diagnosis of LTBI and the difficulties in the interpretation of TST results, especially among immunocompromised patients, strongly contributed to our decision to consider risk as the main criterion for LTBI diagnosis. The classification as high or low risk for LTBI is rather arbitrary, nevertheless it was based upon clinical and epidemiological data. Additionally, we applied a scoring system to facilitate risk definition and considered the traditional concept of contact, following the definition provided by Rose [20].

In our study, we identified significantly discordant results between TST and IGRA, as it was previously shown by other authors [13]. The World Health Organization (WHO) studies concluded that TST may be inadequate for diagnosing LTBI in immunocompromised individuals due to HIV infection [16]. Furthermore, previous studies have also shown that the chronic state of immunosuppression in patients with HIV may lead to false TST negative results, while giving some advantage to the IGRA test, as the latter seems to suffer less influence from low CD4+ T cell counts than the TST [16]. Additionally, in immunocompromised individuals false-negative TST results may occur because late immunosuppression is directly related to T cell activities. Even though CD4+ T cell counts affect the IGRA results, this test seems to be more reliable than the TST for detecting LTBI in this population [27–31]. Another possible explanation for the discrepancy observed in the HIV patient is that the TST result may be negative within two to eight weeks after infection with TB due to a delayed-type hypersensitivity. On the other hand, it is believed that the conversion of the IGRA occurs much earlier after the infection [32].

We found no significant association between the TST and LTBI risk but rather, there were a strong association between the IGRA results and the risk of LTBI in these patients. In fact, when compared to TST, IGRA was more sensitive and specific to identify patients with LTBI. However, the specificity of IGRA observed in our study was still lower than that found in other studies (> 90%), which may reflect some intrinsic characteristics of the Brazilian population [29–31, 33]. According to our results, a negative IGRA test in the person with HIV strongly excluded LTBI. By contrast, those with positive IGRA were more likely to have LTBI. Our data suggests that, when screening HIV-infected persons for LTBI, IGRA may be a better choice than TST.

We are aware that our study presents some limitations, including the important lack of a universally accepted definition of risk of LTBI. Indeed, stratifying patients for high and low risk for LTBI is not an easy task, even with application of a scoring system and following the recommendations previously published [20]. Nevertheless, we were able to show that IGRA detected LTBI with greater reliability, especially among patients presenting lower levels of CD4 + T lymphocyte count. Another limitation is the transversal design of our study, making impossible for us to follow up patients thus assessing the possible association between positive tests and progression to TB disease. Furthermore, TST and IGRA were performed only once for each HIV patient, hindering the possibility of evaluating future tests conversion or even reversal particularly among those who had shown discordant results between tests, which could have clarified the agreement between these tests with the diagnosis of LTBI. We also argue that a longitudinal study using the TST and IGRA for the diagnosis of LTBI would better answer this question and perhaps explain the greater agreement between IGRA with diagnosis of LTBI.

Unfortunately, the definition universally accepted for diagnosing LTBI does necessarily include the performance of TST or IGRA test, which indeed may have biased our results. One attempting to assess the accuracy of a specific test for a defined disease or clinical situation, should not include the same test in the criteria for the definition. Nevertheless, our results showed a strong association between positive IGRA results and the high risk for LTBI in the HIV-positive population included in the study. Moreover, among those HIV-positive patients with lower CD_4_ count, IGRA accuracy was superior when compared to TST. This may represent a strong association between IGRA and epidemiological risk factors itself [3, 8]. In this context, a recent Brazilian Ministry of Health statement recommended that for those HIV-positive patients with CD_4_ count equal or lower than 350 cels/mm^3^, with a defined epidemiological high risk for LTBI and when TB disease was absent should receive therapy regardless of TST or IGRA test results [8, 34]. On the other hand, World Health Organization (WHO) recently recommended that preventive TB treatment should be offered for all HIV-positive patients unlikely to have active TB with unknown or positive TST, regardless of any CD_4_ count [8].

Based on these results, we may speculate that following a clinical evaluation and categorization of risk, individuals with a normal chest radiography with no signs or symptoms of TB disease should undergo IGRA. Upon positivity of IGRA, patient should start treatment as soon as possible. When IGRA is negative, LTBI would be ruled out. For the diagnosis of LTBI, the risk of infection with TB should always be considered. In developing countries with high TB-HIV coinfection burden, and limited public financial resources, TST must be considered the first choice for LTBI diagnosis due to its low cost and ease of performance.

## Conclusions

In the detection of latent tuberculosis infection (LTBI) in HIV-infected individuals, IGRA showed a better performance than the TST and a better association with the risk of *M. tuberculosis* infection. Taken together, our study demonstrated that IGRA has a better performance than the TST for diagnosing infection by TB in HIV-infected individuals. Nevertheless, the challenge for a new diagnostic tool such as IGRA to become a gold standard for LTBI detection in patients with HIV.

## Additional file

Additional file 1: **Figure S1.** ROC curve for IGRA according to the risk of infection with TB in HIV-infected patients at risk of LTBI. (DOCX 134 kb)

## Abbreviations

AIDS: Acquired immunodeficiency syndrome
ART: antiretroviral therapy
BCG: Bacillus Calmette-Guérin
CD4: Cluster of differentiation-4
CD8: Cluster of differentiation-8
CI: confidence intervals
HIV: Human Immunodeficiency virus
IGRA: Interferon Gamma Release Assay
INF-γ: Interferon-Gamma
LTBI: Latent Tuberculosis Infection
NPV: Negative predictive value
PPV: Positive predictive value
S: Specificity
Se: Sensitivity
TB: Mycobacterium Tuberculosis
TB: Tuberculosis
TST: Tuberculin Skin Test
