# Supplementary figures and images for: Interferon-γ release assay as a sensitive diagnostic tool of latent tuberculosis infection in patients with HIV: a cross-sectional study
Source: BMC Infect Dis. 2018 Nov 19;18:585. doi: 10.1186/s12879-018-3508-8 (PMC6245697; doi:10.1186/s12879-018-3508-8)

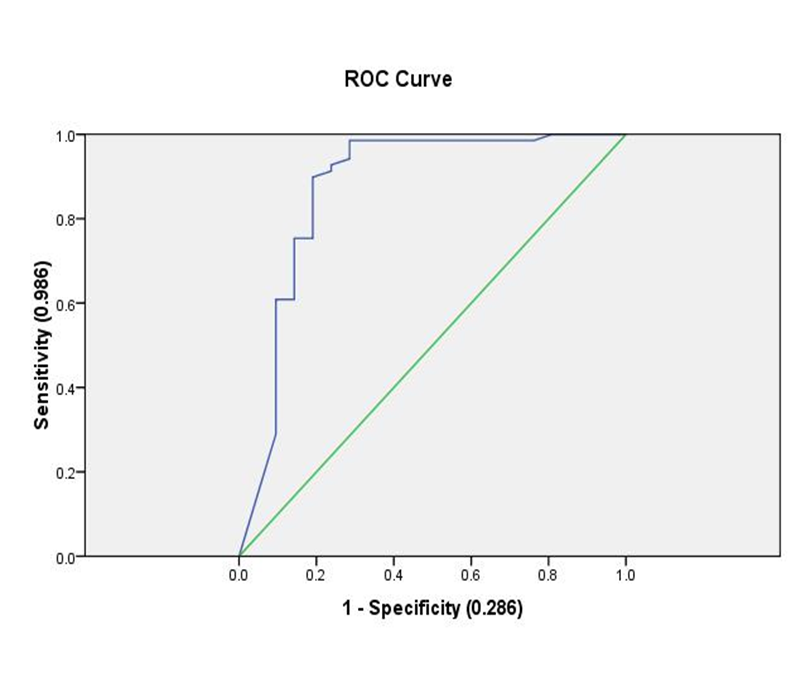


Additional file 1: Figure S1

Supplement: Supplementary file 1 — Figure S1. ROC curve for IGRA according to the risk of infection with TB in HIV-infected patients at risk of LTBI. (DOCX 134 kb) [file 12879_2018_3508_MOESM1_ESM.docx]
